# Are prophylactic antibiotics required for combined intracavitary and interstitial brachytherapy of gynecologic cancers?

**DOI:** 10.1093/jrr/rrae018

**Published:** 2024-04-12

**Authors:** Takuya Kumazawa, Yu Ohkubo, Keishiro Mochida, Saori Kondo, Osamu Oguchi, Daisaku Yoshida

**Affiliations:** Department of Radiation Oncology, Saku Central Hospital Advanced Care Center, 3400-28 Nakagomi, Saku-shi, Nagano 385-0051, Japan; Department of Radiation Oncology, Gunma University Graduate School of Medicine, 3-39-15 Showa-machi, Maebashi, Gunma 371-8511, Japan; Department of Radiation Oncology, Saku Central Hospital Advanced Care Center, 3400-28 Nakagomi, Saku-shi, Nagano 385-0051, Japan; Department of Radiation Oncology, Saku Central Hospital Advanced Care Center, 3400-28 Nakagomi, Saku-shi, Nagano 385-0051, Japan; Department of Obstetrics and Gynecology, Saku Central Hospital Advanced Care Center, 3400-28 Nakagomi, Saku-shi, Nagano 385-0051, Japan; Department of Obstetrics and Gynecology, Saku Central Hospital Advanced Care Center, 3400-28 Nakagomi, Saku-shi, Nagano 385-0051, Japan; Department of Radiation Oncology, Kanagawa Cancer Center, 2-3-2 Nakao, Asahi-Ku, Yokohama, Kanagawa 241-8515, Japan

**Keywords:** gynecological cancer, image-guided brachytherapy, intracavitary and interstitial brachytherapy (IC/IS), hybrid brachytherapy, prophylactic antibiotics

## Abstract

The purpose of this study is to evaluate the need for prophylactic antibiotic treatment prior to combined intracavitary and interstitial (hybrid) brachytherapy for gynecologic cancer. A total of 105 gynecologic cancer patients received 405 brachytherapy sessions, including 302 sessions of intracavitary brachytherapy and 103 sessions of hybrid brachytherapy. Prophylactic antibiotics were administered before 35% of the hybrid brachytherapy sessions. The incidence of postbrachytherapy fever and the frequency of subsequent antibiotic use for infection were compared between treatment groups. Among patients treated with hybrid brachytherapy, fever ≥37.5°C occurred in 16.4% of those not receiving prophylactic antibiotics and 16.7% of those receiving prophylactic antibiotics (*P* > 0.05). Similarly, fever ≥38.0°C occurred in 4.9% of patients not receiving prophylactic antibiotics and 2.4% of those receiving prophylactic antibiotics (*P* > 0.05). Additional antibiotics were used to treat postbrachytherapy infections in 4.8% of the group receiving prophylactic antibiotics and 0% of those not receiving prophylactic antibiotics, again without statistically significant difference. There were also no significant differences in posttreatment fever incidence and antibiotics use for infection between intracavitary brachytherapy and hybrid brachytherapy sessions. In conclusion, the incidences of infection and fever are low following hybrid brachytherapy, so prophylactic antibiotics are generally unnecessary.

## INTRODUCTION

Brachytherapy guided by computed tomography (CT) or magnetic resonance imaging (MRI) is used extensively for the treatment of gynecologic cancers [1–5]. The widespread use of this image-guided approach has also allowed for the development of a hybrid brachytherapy combining intracavitary and interstitial brachytherapy. While interstitial brachytherapy requires a high level of skill, hybrid brachytherapy is a simple method that uses a few interstitial needles in combination with regular intracavitary applicators such as tandem/ring, ovoid and cylinder [6]. Several studies have reported favorable clinical outcomes using hybrid brachytherapy, suggesting its potential as a therapeutic modality for locally advanced cervical cancer [7, 8]. Novel hybrid applicators for combined intracavitary and interstitial brachytherapy have also become available and their usefulness confirmed, suggesting that hybrid brachytherapy will become increasingly popular [9, 10].

However, the risk of infection may be higher following hybrid brachytherapy compared with conventional intracavitary brachytherapy as the former involves inserting needles into the uterine cervix. In Japan, a consensus guideline by the Japan Society for Surgical Infection (http://www.gekakansen.jp/antimicrobial-guideline.html) exists regarding the use of antibiotics to prevent perioperative infections. For example, the guideline recommends prophylactic antibiotic for conization, but not for transurethral tumor resection, where the risk of surgical site infection is low. However, there is no recommendation for the use of prophylactic antibiotics for treatments that are more minimally invasive than interstitial irradiation, such as hybrid brachytherapy. Several studies have examined the use of prophylactic antibiotics in cases where the applicator has caused uterine perforation [11–13], but there is no standard criteria for administration, with some studies reporting use according to individual patient factors such as preprocedural infection (including pyometra) and others according to institutional practices [14, 15]. The judicious use of antibiotics is of course essential to prevent allergies and the development of drug-resistant bacteria [16, 17]. Unfortunately, to the best of our knowledge, there are no studies on the efficacy of prophylactic antibiotics for hybrid brachytherapy.

The purpose of this study is to evaluate the frequency of infections associated with hybrid brachytherapy and to verify the need for prophylactic antibiotics.

## MATERIALS AND METHODS

### Patients characteristics

Consecutive patients with gynecologic cancer (*n* = 105), including cervical, endometrioid, vaginal and vulvar cancers, receiving brachytherapy at our hospital from June 2014 to April 2021 were included in the analyses. Patient characteristics are summarized in Table 1. The uterine cervix was the primary site in 91.4% of these cases. Fifteen patients (14.3%) were diagnosed with pyometra by MRI prior to the first brachytherapy session.

**Table 1:** Patient characteristics

			Patients (*n* = 105)
Age, median years (range)			62 (32–87)
Primary site			
Uterine cervix		Total	96 (91.4%)
	FIGO (2008)	IB1	3 (2.9%)
		IB2	1 (1.0%)
		IIA1	10 (9.5%)
		IIA2	9 (8.6%)
		IIB	43 (41.0%)
		IIIA	5 (4.8%)
		IIIB	11 (10.5%)
		IVA	6 (5.7%)
	Salvage treatment		8 (7.6%)
Uterine corpus			6 (5.7%)
Vagina			2 (1.9%)
Vulva			1 (1.0%)
Comorbidity			
	Diabetes		8 (7.6%)
	Heart disease		3 (2.9%)
	Cerebrovascular disease		5 (4.8%)
Pyometra before brachytherapy^a^			15 (14.3%)
Performance status			
		0	64 (61.0%)
		1	30 (28.6%)
		2	7 (6.7%)
		3	3 (2.9%)
		4	1 (1.0%)

^a^Diagnosis based on MRI taken prior to the first course of brachytherapy.

### Treatment

All patients received 3D image-guided brachytherapy with high-dose-rate (HDR) 192-Ir (microSelectron HDR, Elekta, Stockholm, Sweden). For treatment planning, all patients underwent CT scans installed in the same room. The Fletcher-Suit Asian Pacific applicator (tandem and/or half-size ovoids) was used for most cases of cervical cancer, the Vaginal Applicator for cases of vaginal infiltration, and the Rotte Endometrial Applicator in cases of endometrial cancer. In cases with bulky and/or asymmetric tumors, the patient was treated with 20-cm Trocar Point Needles inserted free hand in addition to the regular intracavitary applicator. All applicator and needle insertions were performed in the lithotomy position. In most cases, interstitial needles were inserted transvaginally, but transperineal insertion was performed in six sessions for two patients. The vagina was disinfected using antiseptic soap (benzalkonium chloride, 0.02%) before inserting the applicator. The tip of the needle inserted into the body was used in a clean manner. No surgical gowns, caps or drapes were used. All treatment details are summarized in Tables 2 and S1. The majority of patients received four brachytherapy sessions, for a total of 405. Of these, 302 were intracavitary brachytherapy and 103 were hybrid brachytherapy sessions.

**Table 2:** Treatment details

Intracavity brachytherapy		Number of sessions (*n* = 302)
Applicator	Tandem and ovoids	242 (80.2%)
	Tandem only	1 (0.3%)
	Ovoids only	17 (5.6%)
	Vaginal cylinder	36 (11.9%)
	Rotte endometrial applicator	6 (2.0%)
Prophylactic antibiotics	Yes	0 (0%)
	No	302 (100%)
Hybrid brachytherapy		Number of sessions (*n* = 103)
Applicator	Tandem and ovoids	69 (67.0%)
	Tandem only	13 (12.6%)
	Ovoids only	2 (1.9%)
	Vaginal cylinder	19 (18.5%)
Number of needles	1	40 (38.8%)
	2	27 (26.2%)
	3	15 (14.6%)
	4	13 (12.6%)
	5	7 (6.8%)
	6	1 (1.0%)
Prophylactic antibiotics	Yes	36 (35.0%)
	No	67 (65.0%)

### Prophylactic antibiotic administration

Prophylactic antibiotics were not administered to any patient receiving intracavitary brachytherapy without concomitant needle insertion, whereas prophylactic treatment with 1-g Cefazolin (CEZ) was administered to all patients receiving hybrid brachytherapy until November 2017. Administration of CEZ started when the patient left the ward and ended before the start of RALS. However, starting in December 2017, prophylactic antibiotic treatment was terminated for the following reasons: (i) temporary instability in the supply of antibiotics necessitated restrictions on antibiotic administration within our hospital, (ii) institutional cases of anaphylactic shock because of antibiotic administration prompted a reassessment of prophylactic antibiotics and (iii) Studies suggesting a low incidence of infections following brachytherapy for prostate cancer even without prophylactic antibiotics [18]. As a result, hybrid brachytherapy sessions were divided into prophylactic antibiotic-treated and untreated groups according to when brachytherapy was administered.

### Efficacy evaluation of prophylactic antibiotics

The evaluated parameters were (i) fever ≥37.5°C, (ii) fever ≥38.0°C and (iii) antibiotic treatment for infection within 1 week after brachytherapy.

### Statistical analysis

The incidence of postprocedural fever and the frequency of later antibiotic treatment for infections were compared by Fisher’s exact test, with *P* ≤ 0.05 (two-sided) defined as significant. All statistical analyses were performed using SPSS Version 28.0.

## RESULTS

To assess the necessity of prophylactic antibiotic treatment for hybrid brachytherapy, we compared the incidence of fever and the later use of antibiotics to treat infections after brachytherapy between intracavity and hybrid brachytherapy groups (Table 3). The incidence of fever ≥37.5°C within 1 week after brachytherapy did not differ significantly between intracavity and hybrid brachytherapy groups (11.9 vs 16.5%, *P* > 0.05). Similarly, the incidence of fever ≥38°C did not differ between groups (2.3 vs 3.9%, *P* > 0.05). In the intracavity brachytherapy group, four patients (1.3%) were administered antibiotics after brachytherapy to treat infections, of which two cases were attributed to intrauterine infection and two cases to pharyngitis. In the hybrid brachytherapy group, only two patients (1.9%) were administered antibiotics after treatment, in both cases for urinary tract infections.

**Table 3:** Incidence of fever ≥37.5°C, incidence of fever ≥38.0 °C and use of antibiotics for treatment of infection after brachytherapy with or without additional needles

Treatment modality	Fever ≥37.5°C		Total	*P* value
	Yes	No		
Intracavity brachytherapy	36 (11.9%)	266 (88.1%)	302 (100%)	0.239
Hybrid brachytherapy	17 (16.5%)	86 (83.5%)	103 (100%)	
Total	53 (13.1%)	352 (86.9%)	405 (100%)	
Treatment modality	Fever ≥38.0 °C		Total	*P* value
	Yes	No		
Intracavity brachytherapy	7 (2.3%)	295 (97.7%)	302 (100%)	0.482
Hybrid brachytherapy	4 (3.9%)	99 (96.1%)	103 (100%)	
Total	11 (2.7%)	394 (97.3%)	405 (100%)	
Treatment modality	Use of antibiotics to treat infections		Total	*P* value
	Yes	No		
Intracavity brachytherapy	4^a^ (1.3%)	298 (98.7%)	302 (100%)	0.647
Hybrid Brachytherapy	2^b^ (1.9%)	101 (98.1%)	103 (100%)	
Total	6 (1.5%)	399 (98.5%)	405 (100%)	

Fisher’s extract test, *P* < 0.05.

^a^Two patients developed intrauterine infection and two developed pharyngitis.

^b^Two patients developed urinary tract infection.

Differences in fever incidence and antibiotics use for infection after brachytherapy were also compared between patient receiving hybrid brachytherapy with or without prophylactic antibiotics (Table 4). The incidence of postprocedural fever ≥38°C did not differ between prophylaxis and nonprophylaxis patient groups (one case or 2.4% vs three cases or 4.9%, *P* = 0.6436). In addition, two cases (4.8%) in the prophylaxis group required additional antibiotic therapy to treat postbrachytherapy infections, whereas no cases in the nonprophylaxis group required antibiotic intervention for infection (*P* = 0.164).

Fifteen patients were diagnosed with pyometra based on prebrachytherapy MRI. These patients received 56 brachytherapy sessions, of which 14 were of hybrid brachytherapy with no prophylactic antibiotics, and no cases of fever ≥37.5°C or infection necessitating antibiotic treatment were encountered. Antibiotic therapy was administered to treat intrauterine infection after brachytherapy in one case with pyometra receiving intracavity brachytherapy.

**Table 4:** Incidence of fever ≥37.5 °C, incidence of fever ≥38.0°C and use of antibiotics for treatment of infection after hybrid brachytherapy with or without prophylactic antibiotics

Prophylactic antibiotics	Fever (≥37.5 °C)		Total	*P* value
	Yes	No		
Yes	7 (16.7%)	35 (83.3%)	42 (100%)	1.000
No	10 (16.4%)	51 (83.6%)	61 (100%)	
Total	17 (16.5%)	86 (83.5%)	103 (100%)	
Prophylactic antibiotics	Fever (≥38.0 °C)		Total	*P* value
	Yes	No		
Yes	1 (2.4%)	41 (97.6%)	42 (100%)	0.644
No	3 (4.9%)	58 (95.1%)	61 (100%)	
Total	4 (3.9%)	99 (96.1%)	103 (100%)	
Prophylactic antibiotics	Use of antibiotics to treat infections		Total	*P* value
	Yes	No		
Yes	2^a^ (4.8%)	40 (95.2%)	42 (100%)	0.164
No	0 (0%)	61 (100%)	61 (100%)	
Total	2 (1.9%)	101 (98.1%)	103 (100%)	

Fisher’s extract test, *P* < 0.05

^a^Two patients developed urinary tract infection.

## DISCUSSION

Prophylactic antibiotic treatment did not significantly reduce the incidence of fever or the need for additional antibiotic administration for infection following hybrid brachytherapy compared with the same brachytherapy procedure performed without prophylactic antibiotics. Therefore, prophylactic antibiotic treatment does not significantly benefit patients receiving hybrid brachytherapy.

Prophylactic antibiotic treatment has been reported to reduce infections following low-dose-rate intracavitary irradiation brachytherapy with radium, but to our knowledge, no report has examined the need for prophylactic antibiotic administration with HDR brachytherapy [19]. Prophylactic antibiotics are not usually used for intracavitary brachytherapy at our institution but are recommended for interstitial brachytherapy of gynecologic cancers [20]. Prostate biopsy with transperineal needle insertion and brachytherapy for prostate cancer are also usually performed with prophylactic antibiotics, but some reports suggest that this treatment is not necessary [18, 21]. Similarly, other reports have suggested that prophylactic antibiotics are unnecessary during excision of the cervical transformation zone, and so should be avoided or used only for clinical research because of the risk of increased antibiotic resistance [22]. Based on these considerations, if the procedure is less invasive, prophylactic antibiotics may be less useful. The current Japanese guidelines for hybrid brachytherapy state that ‘Antibiotics may be considered if there is a retained pyometra and postoperative infection is highly anticipated or if a postoperative infection has occurred during previous treatment’ [15]. According to these guidelines, prophylactic antibiotic administration is not mandatory, although no evidence has been presented showing that such treatment is of no benefit. The current results indeed suggest that hybrid brachytherapy, which is less invasive than interstitial brachytherapy, can be safely performed without prophylactic antibiotic treatment.

To *et al.* reported that 67.74% of patients developed fever ≥37.5°C and 20.43% developed fever ≥38°C following intracavitary radium insertion without prophylactic antibiotics, and that prophylactic antibiotics reduced the incidence of fever ≥37.5°C to 41.93% and fever ≥38°C to 8.60% [18]. They also reported that prophylactic antibiotics significantly reduced the need for additional antibiotics to treat subsequent infections from 13.98 to 4.30% of cases. In our present study, fever ≥37.5°C was observed in 11.9% of cases following intracavitary brachytherapy and in ~16% following hybrid brachytherapy regardless of prophylactic antibiotic use, whereas fever ≥38°C was observed in only 2.7% of all cases. The lower incidence of fever reported here may be because of the smaller-diameter applicator used compared with the intracavitary radium insertion applicator used by To *et al*. as well as the shorter duration of applicator insertion. In six cases with fever ≥38°C (including three each with and without prophylactic antibiotics), no clear infectious focus was identified by blood culture or other test, and the fever resolved spontaneously without antibiotics. Therefore, it is possible that some cases of fever were physiological responses to the invasive brachytherapy procedure. However, six cases (1.5%) did require antibiotic therapy after brachytherapy for intrauterine infection, pharyngitis or urinary tract infection. Based on the results of this study, prophylactic antibiotics are not considered necessary; however, if fever is observed after brachytherapy, the patient should be thoroughly evaluated for infection and antibiotics administered as appropriate.

One notable limitation of this study is that the majority of cases were treated by transvaginal insertion, whereas only two cases received transperineal insertion. In the two cases of transperineal insertion, prophylactic antibiotics were administered and there was no posttreatment fever or infectious complications requiring additional antibiotic therapy. Thus, the possibility that prophylactic antibiotic therapy may have been effective in these transperineal cases cannot be excluded, and further study is warranted.

In conclusion, this study investigated the need for prophylactic antibiotics during hybrid brachytherapy. Approximately 16% of patients receiving hybrid brachytherapy developed fever ≥37.5°C with or without prophylactic antibiotics, whereas only a small number of patients not receiving such treatment developed high fever ≥38°C. Furthermore, there were few cases requiring antibiotic therapy after brachytherapy conducted without prophylactic antibiotics. The use of prophylactic antibiotics may be considered in cases with a high risk of infection, but in general, prophylactic antibiotic treatment is unlikely to benefit patients receiving hybrid brachytherapy. However, since the data presented here are retrospective and from a single center, a prospective randomized clinical trial is needed to confirm these results.

## Supplementary Material

231222Table_S1_rrae018

## PRESENTATION AT A CONFERENCE

Parts of this study were presented at the 34th Annual Meeting of the Japanese Society for Radiation Oncology, Online, 12–14 November 2021.
