# Supplementary material for: Are prophylactic antibiotics required for combined intracavitary and interstitial brachytherapy of gynecologic cancers?
Source: J Radiat Res. 2024 Apr 12;65(3):387–92. doi: 10.1093/jrr/rrae018 (PMC11115469; doi:10.1093/jrr/rrae018)
Supplement: 231222Table_S1_rrae018 [file 231222table_s1_rrae018.docx]

Table S1. Treatment details in hybrid brachytherapy

|  |  |  | Prophylactic antibiotics | |
| --- | --- | --- | --- | --- |
|  | | All (n = 103) | Yes (n = 36) | No (n = 67) |
| Applicator | Tandem and Ovoids | 69 (67.0%) | 20(55.6%) | 49(73.1%) |
|  | Tandem only | 13 (12.6%) | 3(8.3%) | 10(14.9%) |
|  | Ovoids only | 2 (1.9%) | 1(2.8%) | 1(1.5%) |
|  | Vaginal Cylinder | 19 (18.5%) | 12(33.3%) | 7(10.4%) |
| Number of needles | 1 | 40 (38.8%) | 6(16.7%) | 34(50.7%) |
|  | 2 | 27 (26.2%) | 12(33.3%) | 15(22.4%) |
|  | 3 | 15 (14.6%) | 5(13.9%) | 10(14.9%) |
|  | 4 | 13 (12.6%) | 8(22.2%) | 5(7.5%) |
|  | 5 | 7 (6.8%) | 4(11.1%) | 3(4.5%) |
|  | 6 | 1 (1.0%) | 1(2.8%) | 0(0%) |
